# A triple test for behavioral economics models and public health policy

**DOI:** 10.1007/s11238-017-9625-9

**Published:** 2017-07-18

**Authors:** Ryota Nakamura, Marc Suhrcke, Daniel John Zizzo

**Affiliations:** 1 Hitotsubashi Institute for Advanced Study, Hitotsubashi University, 2-1 Naka, Kunitachi, Tokyo 186-8601 Japan; 2 Centre for Health Economics, University of York, Heslington, York, YO10 5DD UK; 3 Faculty of Humanities and Social Sciences, Newcastle University, 5th Floor, Daysh Building, Newcastle upon Tyne, NE1 7RU UK; 4 BENC and Newcastle University Business School, Newcastle upon Tyne, UK

**Keywords:** Behavioral economics, Nudges, Peer effects, Self-control, Prospect theory, Framing effect

## Abstract

We propose a triple test to evaluate the usefulness of behavioral economics models for public health policy. Test 1 is whether the model provides reasonably new insights. Test 2 is on whether these have been properly applied to policy settings. Test 3 is whether they are corroborated by evidence. We exemplify by considering the cases of social interactions models, self-control models and, in relation to health message framing, prospect theory. Out of these sets of models, only a correctly applied prospect theory fully passes the tests at present. Specifically, in broad agreement with the evidence, a gain frame has positive implications for welfare encourages disease prevention activity, though this does not apply if the perceived probability of the bad health outcome is large enough. We see our tests as being useful to identify how much health policy weight policy makers should assign to specific behavioral economic models; they are also useful to verify what next steps would be most useful in further research.

**Electronic supplementary material:**

The online version of this article (doi:10.1007/s11238-017-9625-9) contains supplementary material, which is available to authorized users.

## Introduction

Behavioral economics (BE) has been seen as holding great promise in a range of policy applications, including that of improving health outcomes (Frank 2004; Zimmerman 2009; Loewenstein et al. 2007, 2012; Barberis 2013). This promise has been recognized by policy- makers across a range of countries, including France (Ouillier and Sauneron 2010), the United States (Lott 2013) and the United Kingdom (Dolan et al. 2010). It has broadly matched the rise of the behavioral ‘nudge’ agenda: the possibility of obtaining quick wins in terms of policy outcomes by altering the decision environment of the individual in a way that does not forbid any option or change any economic incentive (see Thaler and Sunstein 2008). The original classic example by Thaler and Sunstein concerned the case of a cafeteria where, by changing the placement of healthy and unhealthy food, it would be possible to affect the extent to which agents chose each. The alleged policy advantages, particularly to policy makers in an age of economic recession, were clear: the potential of better health outcomes without restricting the choice set of the rational consumer and, significantly, at little or no cost for the policy maker.^1^

That said, a disconnection between the excitement of the promise of BE and the evidence base has been noted (Marteau et al. 2011). Early proposers of BE have put this in terms of policy getting ahead of science (Loewenstein et al. 2012), and of hard shoves (in terms of regulation) being needed as much as soft nudges. A recent report of the U.K. House of Lords has reached qualified conclusions on the potential of using only behavioral interventions in affecting outcomes (House of Lords 2011). A recent scoping review of choice architecture interventions has reached the conclusion that the jury is still out on effect sizes for such interventions, both singly and in combination (Hollands et al. 2013).

The key question of this paper is the degree to which BE is *actually* adding to the public health policy debate. For the purpose of this paper, we define BE as comprising economic models that relax the standard assumptions of rationality, pure self-interest or both. We label ‘standard economic models’ as models that keep both of these assumptions. BE mostly encapsulates and incorporates concepts and findings from psychology or cognate disciplines, which are combined with economic modelling to produce potentially *new insights* hopefully of interest outside economics, including to policy makers (for examples, see Camerer et al. 2004; Skořepa 2011; Cartwright 2011). We propose a triple test for whether a behavioral economic model is relevant for public health policy:

- *Test *1: the model has to yield specific predictions relative to standard economic models or established psychological theories in terms of individual behaviors or reaction to incentives;
- *Test *2: the model has to provide specific predictions regarding specific public health policies;
- *Test *3: the model has to be appropriately validated by empirical evidence.

This paper considers example BE models and shows how these tests can be usefully employed.^2^ We consider three areas where one can, with some legitimacy, claim that the first test is passed: social interactions; self-control devices; and prospect theory. We find that, with the partial possible exception of the area of self-control, in all three areas there needs to be further progress on the connection between insights from BE models, policy application and corroboration. We suggest that the proposed triple test can be employed to verify the policy relevance of BE insights. Section 2 provides the conceptual background to our triple test. Sections 3, 4 and 5 consider our three areas of application. The supplementary material provides relevant lists of empirical references and key findings. Section 6 concludes.

**Fig. 1:**
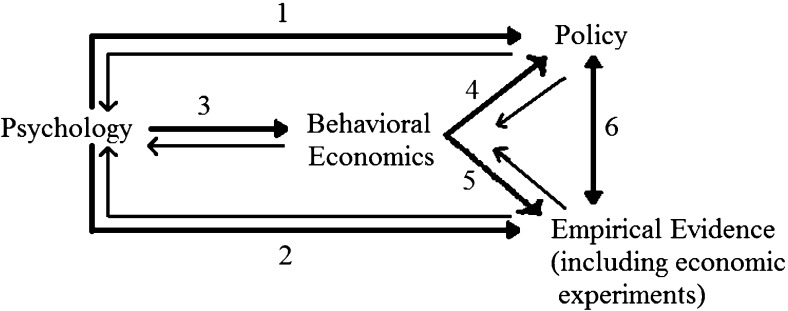
Identifying the value added of behavioral economics

## A conceptual background

Figure 1 helps clarify key points underpinning the triple test being proposed here. There is a long tradition of psychological research having policy implications (link 1 in Fig. 1); there is also a sizeable amount of empirical evidence in connection to psychological concepts, including economic experiments that connect to them (link 2). While this needs not always be the case, BE often employs economic modelling to formalize concepts and findings from psychology and deviations from standard economic models (link 3); for example, the notion of social comparison and relative utility which we shall consider in Sect. 3 draws its parentage both on social psychology, e.g., the social exchange theory of Adams (1963), and on the cognitive psychology of relative evaluations (e.g., Kahneman and Varey 1991, for references). BE can have implications for policy (link 4). *Test 1* is about whether something is gained conceptually in moving from psychology to policy through links 3 and 4 rather than directly via link 1. In other words, does a behavioral economic model provide any reasonably original insight that one would not be able to glean by employing not just standard economic models but also plain vanilla psychological concepts? Note that we are not stating that BE must not relate to or be inspired by psychological models. Clearly, this will typically be the case. Nevertheless, the answer to the question on whether original insights are provided will not always be positive, for two reasons.

First, some of BE has been about formalizing psychological concepts in rational choice models that do not add any particular insight relative to such concepts, at least in relevant policy domains. For example, Akerlof and Kranton (2000) add a utility function but little more to the kind of insights that can be drawn from the social psychological research on group identity and intergroup relations (e.g., Hogg and Abrams 2001, for a review). While this exercise is deemed valuable by economists insofar as utility maximization is considered as the methodological golden rule by most economists, non-economists and policy makers may not learn anything more than they would by referring directly to the appropriate psychological concepts.

Second, sometimes BE is used to refer to concepts taken straight from traditional behavioral psychology; for example, Murphy et al.’s (2007) review speaks of behavioral economic approaches to reduce college student drinking, but actually simply speak in terms of relative reinforcement and in terms of traditional economics (law of demand), neither of which require any behavioral economic models; there is nothing in Murphy et al. (2007) that modern behavioral psychological treatments (e.g., Fantino and Logan 1979; Rachlin 1989) would not be able to explain.

*Test 2* is also connected to link 4, and is about whether specific predictions follow from the model for specific public health contexts; as Sect. 5 on prospect theory will illustrate, this is not always straightforward.

Evidence-based policy requires, however, that in order for a prediction from BE to be relevant, it not only has to be accurately drawn from a given behavioral economic model for a specific public health setting, but also it has to be supported by evidence. *Test 3* is connected to links 5 and 6 and requires evidence of validation of a given behavioral economic model. Obviously, the extent to which it is possible to test predictions that unambiguously validate a model may depend on the type of evidence available, whether in the form for example of randomized control trials, laboratory or field experiments, or econometric studies. Also, in considering evidence, there is no reason to be restricted to evidence from economics (let alone behavioral and experimental evidence).

One source of confusion with empirical evidence is that sometimes empirical studies are motivated by policy (link 6) rather than by theory. As such, they are not tailored to test specific behavioral economic models, and as a result they provide only weak evidence in the context of Test 3; Sect. 4.3 will show an example of this.

## Applying social interactions models to health behavior

### Social interactions models and test 1

Our first illustration arises from research in BE on how peers’ behavior influences one’s behavior, which can be labelled as *peer effects *for short. We consider three possible channels through which peer effects have been modelled to affect health behavior: (i) social learning; (ii) social comparison; (iii) self-esteem or moral concerns.

*Social learning* refers to the idea that what others do has information that is relevant for one’s choices, particularly when she is uncertain about the (health) consequence of her behavior (Bikhchandani et al. 1992). The inspiration for this comes from social learning theory in psychology, which has a long tradition (e.g., Bandura et al. 1961; Bandura 1977).

*Social comparison* models reflect the idea that preferences are shaped by comparisons of oneself with others leading to conformism. The average behavior in the society provides a reference point, and deviating from the reference point decreases one’s utility (Blanchflower et al. 2009). A slightly different way to look at the social comparison motive is that conformity may construct peer ties, which itself can be desired (i.e., social capital).

In *self-esteem, moral and social scrutiny *models, a norm level of behavior is exogenously given in a social group, and an individual loses utility if he or she is seen as deviating from the norm. For example, Dragone and Savorelli (2012) investigate the effect of manipulating the norm level of body shape by legislations such as banning underweight fashion models. Of course, social psychologists have long recognized the significance of social interactions (e.g., Asch 1955; Bond and Smith 1996). These BE models potentially add specific policy relevant predictions. For example, and following Dragone and Savorelli (2012), public health marketing policies to reduce the risk of anorexia may lead to negative health costs in terms of promoting obesity that more than offset the health benefits. Because of this, there is at least the potential for Test 1 to be passed, i.e., for insights to be provided that would not just be gleaned from non-BE research.

### Combining motivations from social interactions models

We present a composite representation which describes how health behavior is influenced by social interactions. Social influence on health behavior including these three motives has been investigated separately in the literature. In this sub-section, we combine the three motives within one simple framework. For simplicity, we do not consider pecuniary motives of health behavior (such as cost of medical treatments). We introduce two types of norms, following Bicchieri (2006) and Bicchieri and Xiao (2009). The first norm is based on empirical expectations, i.e., the observation of peers’ actual behavior. The second norm is based on normative expectations, i.e., the observation of peers’ desire or what peers expect him or her (not) to behave. These two norms are often distinctive in health contexts. For example, there are people who smoke when they observe their peers smoking, but share the idea that they should not smoke.

Consider a person j’s health behavior $x_\mathrm{j}$, $x_{-\mathrm{j}}^{E}$ denoting the empirical expectation of peers’ behavior, and $x_{-\mathrm{j}}^{N}$ capturing the normative expectation. The individual maximizes the following utility by choosing the optimal $x_\mathrm{j}$:

$$\begin{aligned} U_\mathrm{j} =-\frac{{\alpha }}{2}\left[ {x_\mathrm{j} -x_\mathrm{j}^0 \left( {x_{-\mathrm{j}}^{E} ,\epsilon } \right) } \right] ^{2}-\frac{{\beta }}{2}\left[ {x_\mathrm{j} -x_{-\mathrm{j}}^{E} } \right] ^{2}-\frac{{\upgamma }}{2}\left[ {x_\mathrm{j} -x_{-\mathrm{j}}^{N} } \right] ^{2} \end{aligned}$$

The utility comprises three distinctive motives, and deviation from them decreases utility. The first term represents the social learning motive, by which $x_\mathrm{j}^0$ is her subjective ideal level of behavior, which is influenced by observation of peers’ actual behavior $x_{-\mathrm{j}}^{E}$ and an idiosyncratic probabilistic component $\epsilon$. This means that the person may not be fully certain about what his or her best choice will be (due to lack of information), and he or she uses peers’ behavior to form her preference (i.e., social learning). The second term of the utility function captures social comparison conformism in the sense that the person sets peers’ behavior as a reference point, and prefers to conform even if it does not meet his or her self-interest motive (i.e., social comparison). Finally, the third part gives self-esteem and moral concern, where the person wants to behave as he or she perceives it is desired by peers $x_{-\mathrm{j}}^{N}$ to avoid disapprovals (as long as ${\beta }, {\gamma }>0)$. Ignoring corner solutions, the first order condition is:

1 $$\begin{aligned} x_\mathrm{j}^{*} =\frac{1}{{\alpha }+{\beta }+{\gamma }}\left[ {{\alpha x}_\mathrm{j}^0 \left( {x_{-\mathrm{j}}^{E} ,\epsilon } \right) +{\beta x}_{-\mathrm{j}}^{E} +{\gamma x}_{-\mathrm{j}}^{N} } \right] \end{aligned}$$

This is simply a weighted average of the subjective ideal, the empirical expectation, and the normative expectation. This implies that the individual’s behavior is determined by the relative importance of three motives. When the self-interest motive is important (i.e., large $${\upalpha })$$, the individual’s behavior is more consistent with her subjective ideal level $$x_j^0 $$. The same logic applies to other cases, i.e., when social comparison is prominent the individual acts as others do; when self-esteem is more important, she behaves as (she thinks) is desired by others.

As shown later, most of the econometric analyses estimate the effect (or association) of empirical expectation $$x_{-j}^E $$ on behavior $$x_j^*$$. Totally differentiating the first order condition Eq. (1) yields:

2 $$\begin{aligned} \frac{\hbox {d}x_\mathrm{j}^{*} }{\hbox {d}x_{-\mathrm{j}}^{E} }=\frac{1}{{\alpha }+{\beta }+{\gamma }}\left[ {{\alpha }\frac{\partial x_\mathrm{j}^0 }{\partial x_{-\mathrm{j}}^{E} }+{\beta }} \right] \end{aligned}$$

This representation of the effect suggests that without an elaborate estimation strategy the analysis does not distinguish the three motives. We return to this point in Sect. 3.4.

### Test 2: policy implications

There are specific policy predictions from models embodying different motivations related to social interactions, and in this sense Test 2 is satisfied. Each motive suggests meaningfully different implications for policies to promote healthy outcomes. If social learning is most prominent, an appropriate policy would be to provide precise information about others’ health behavior, and also the consequence of the behavior, through health educational policies. There is evidence that over-estimation of peers’ smoking rate is a significant determinant of smoking among adolescents (Reid et al. 2008). Also, if the reason for conformity is uncertainty in preference, labelling and setting a default option in favor of healthier behavior would be helpful (Wisdom et al. 2010).

If social comparison is important, giving information about the “right” behavior will not work, because individuals follow peers irrespective of how healthy or unhealthy the behavior may be. A possible policy would then require nicotinizing a shift to a healthier behavior equilibrium. For example, the government or schools can increase punishment for youth smoking. The government could also subsidize healthier options such as gym charge.

If self-esteem, moral or social scrutiny is a main driver of peer effects, a less resource-intensive policy could be effective to manipulate the perceived norm. For example, media has strong power on one’s perception over what others think desirable. Therefore, campaigning to change normative expectation through media, or restricting the exposure to unhealthier norms, may prove to be an effective intervention.

### Test 3: empirical evidence

Test 3 requires us to find evidence able to corroborate the specific predictions associated with the different motivations associated with models linked to different motivations.

*Experimental evidence*. Interventions through social interactions to health behavior have been mainly outside the economics literature. Most of such experimental studies examine information-giving type interventions. For example, in a laboratory, providing information about norms, such as others’ attitude towards food and actual food consumption, can influence own behavior (Croker et al. 2009; Pliner and Mann 2004). Also, providing web-based and face-to-face feedback about norms can reduce alcohol misuse among college students (Moreira et al. 2009). While evidence of the effectiveness of providing information seems most consistent with a social learning story, it could equally be consistent with the other two motivations: it could provide information relevant for social comparison and, in a social scrutiny perspective, it could make clear what the norm is and indeed what the experimenter wants experimental subjects to do (Zizzo 2010). Zafar’s (2011) experiment usefully tries to decompose social comparison and social scrutiny motives on charitable behavior. Image concern is controlled by restricting the observability of one’s donating behavior, so that there is no chance to earn esteem. The result indicates that both motives effectively influence behavior. However, the experiment is not in a health context and does not control for social learning.

*Non-experimental evidence*. Non-experimental studies in this particular field—perhaps understandably—do not appear to be well connected to theoretical implications. So far the main purpose of the econometric studies has been to identify the causal effect of peers’ behavior on one’s own behavior, and this is where (behavioral) economists have made major methodological contributions to the literature. However, so far the studies typically do not fully address the motives underlying peer effects. See Table A1 in the Online Appendix for a list of 36 relevant econometric and experimental studies. Out of these, 15 studies investigate the peer associations in food consumption and body weight; 17 studies are on substance use (in particular tobacco and alcohol); 4 are studies on other outcomes (e.g., healthcare plan choice, sick leave). The definition of social group (peers) varies remarkably from broader level (e.g., same sex, same country) to narrow levels (neighborhood, classmates, roommates and close friends). Some studies build theoretical models (as shown in the previous subsection), or explicitly mention some background theoretical implications to motivate their empirical investigations.

The interpretability of regression coefficients is a challenging point for econometric studies. A simple statistical association between peers behavior and one’s own behavior may not reveal peer effects (Manski 1993, 2000). First, peers’ behavior influences one’s behavior, and vice versa. Second, unobservable factors may be correlated with both peers’ behavior and own behavior. Some previous studies present various strategies to control for these confounding effects (for example, Nakajima 2007; Krauth 2006; Lee 2007). However, even when a researcher successfully controls for these confounds, the question remains about *why* there are the peer effects in health behavior. As shown by Eq. (2) and our earlier discussion, establishing a peer effect is a necessary but not a sufficient condition to corroborate a specific health-related behavioral motivation and its policy implications, since it does not distinguish possible motives to conform. In this sense, there exists another fundamental identification problem.

### Summary

The domain of social interactions is an illustration where BE models hold potential for Tests 1 and 2, but there is an identification gap between models and evidence. While enough evidence can be conjured that social interactions in some sense matter, there is an insufficiently sharp evidence base from *specific *motivations connected to social interactions motives. Therefore Test 3, on evidence corroborating models and policies, is a stumbling block.

## Applying self-control models to health behavior

### Self-control devices models and Test 1

In psychology, self-control is typically seen as part of self-regulatory control processes (e.g., Carver and Scheier 1982) or as response inhibition in the context of modern neuropsychological models of behavior (Diamond 2013). Within BE, self-control models encapsulate the basic game-theoretical intuition that fewer options can be good by working as a commitment device. Because of the natural way in which this research stems from this intuition from economics rather than psychology, the case for satisfying Test 1 is reasonably straightforward, and specific policy predictions follow from these models in health contexts to support this. Self-control devices can work as commitment devices to promote positive health outcomes (Bryan et al. 2010).

Three modeling approaches to self-control problems are choice-set utility (Gul and Pesendorfer 2001), intertemporal choice (Bernheim and Rangel 2004) and multiple selves (Gruber and Köszegi 2001). While these models embody a similar intuition, they sometimes draw different policy implications. For example, on the one hand studies employing a hyperbolic discounting function generally suggest the use of fiscal interventions such as tax to manipulate the price of tempting goods (Gruber and Köszegi 2001). On the other hand, Bernheim and Rangel (2004) predict that a price increase may not discourage consumption of tempting goods when self-control is restricted in the hot state of mind. They suggest that avoiding environmental stimuli that drive individuals to impulsive behavior may be a potential solution to address self-control problem. While the specificity of these predictions ensures that Test 1 is passed, they create potential problems of identification of the kind we discussed in the context of social interactions.

### An illustrative model and Test 2

This section presents a simple model of self-control problems based on quasi-hyperbolic discounting (Phelps and Pollak 1968). For illustrative purpose, we consider a smoking decision. Similarly to O’Donoghue and Rabin (2006), we assume that smoking gives positive immediate utility and affects health next period. Let *S* denote smoking, which takes 1 if the person smokes and 0 otherwise. The immediate utility of smoking is represented by $v\left( S \right)$, and the health damage is represented by $-h\left( S \right)$, where we assume $v\left( 0 \right) =h\left( 0 \right) =0$. The cost of smoking is *p*. The rest of the person’s instantaneous budget *M* is spent on the composite good (which gives linear positive utility) and the price is normalized to 1. Finally, we assume that the intertemporal utility is given by the following standard quasi-hyperbolic discounting formulation:

$$\begin{aligned} u_0 +\beta \sum \limits _{k=1}^T \delta ^{k}u_k , \end{aligned}$$

where $\beta$, $\delta \le$1. The parameter $\beta$ is often interpreted as the degree of self-control problem. If $\beta =1$, the function is the usual exponential discounting function.

For simplicity, we consider three periods (t=0, 1 and 2). In period 0, the person plans whether or not to smoke in period 1; then, in period 1, the utility from smoking materializes; and finally, the health damage of smoking is incurred in period 2. In period 0 the person just plans (i.e., *planner*), and the actual behavior is taken in period 1 (i.e., *doer*). From the planner’s perspective in period 0, the utility in period 1 is given by $\beta \delta \left[ {v\left( 1 \right) +M-p} \right]$ if she smokes, and $\beta \delta M$ if she does not smoke. Also, the utility in period 2 is given by $\beta \delta ^{2}\left[ {-h\left( 1 \right) } \right]$ if he or she smokes in period 1 and 0 otherwise. The planner decides to smoke in period 1 if the net utility of smoking exceeds the net utility of non-smoking: $\beta \delta \left[ {v\left( 1 \right) +M-p} \right] +\beta \delta ^{2}\left[ {-h\left( 1 \right) } \right] \ge \beta \delta M$. Hence, the person plans to smoke if:

$$\begin{aligned} p\le v\left( 1 \right) -\delta h\left( 1 \right) . \end{aligned}$$

For the planner, the reservation price of a cigarette is given by $p^{*}=v\left( 1 \right) -\delta h\left( 1 \right)$. In period 0, the person plans to smoke in period 1 if the cost of smoking is lower than the immediate utility of smoking minus the discounted future health damage.

We turn to consider the *doer*’s problem in period 1. The immediate utility of smoking is given by $v\left( 1 \right) -M-p$ if she smokes, and *M* if she does not. The utility in the next period is given by $\beta \delta \left[ {-h\left( 1 \right) } \right]$ if she smokes, and 0 if she does not. The individual smokes in period 1 if $v\left( 1 \right) -M-p+\beta \delta \left[ {-h\left( 1 \right) } \right] \ge M$. The doer smokes if:

$$\begin{aligned} p\le v\left( 1 \right) -\beta \delta h\left( 1 \right) . \end{aligned}$$

The doer’s reservation price is given by $$p^{**}=v\left( 1 \right) -\beta \delta h\left( 1 \right) $$. Compared to the previous condition to smoke for the planner, the doer discounts the future health damage more heavily by $$\beta \delta $$ (where $$\beta $$, $$\delta \le $$1). This means that the doer accepts a higher cigarette price than the planner. More specifically, if the actual cigarette price is between $$p^{*}$$ and $$p^{**}$$: $$v\left( 1 \right) -\delta h\left( 1 \right)<p<v\left( 1 \right) -\beta \delta h\left( 1 \right) $$, the doer smokes in period 1 even though he or she planned not to smoke, a *preference reversal.*

When this preference reversal is likely to happen, there are ways for the planner to restrict the doer’s behavior. The planner should make the doer’s reservation price of cigarette ($$p^{**})$$ closer to the planner’s original reservation price $$p^{*}$$. Stronger restriction will be needed depending on the degree of the self-control problem: a smaller $$\beta $$ (i.e., larger discount) implies the need for stronger restrictions.

Commitment helps restrict the doer’s behavior. For instance, the planner can commit to pay a higher price for a cigarette in period 1, so that the doer faces the higher price. Similarly, he or she can commit to paying some amount of money in case she smokes. Both commitments directly decrease the reservation price of cigarette for the doer $$p^{***}=v\left( 1 \right) -\beta \delta h\left( 1 \right) -C$$, where *C* is either the increased price or the punishment. Incurring additional cost to smoke in this way brings the doer’s reservation price closer to $$p^{*}$$.

Test 2 is met, in the sense that specific predictions for public health contexts follow from these models. Some interventions can be regarded as self-control devices of a similar kind. For example, fiscal interventions, such as tax or income transfer, can alter the reservation price for the doer in the same way as the self-commitment. As in O’Donoghue and Rabin (2006) and elsewhere, exercising a higher tax on cigarettes may prevent self-control lapses. Rewarding individuals for not smoking by lump-sum transfer will work in the same direction as the price intervention. Immediate rewards may be more effective, if the individual discounts the future rewards heavily (Loewenstein et al. 2007).

Sometimes commitments may be considered as not involving (only) pecuniary incentives but, for example, may include social image costs. In the above example, the (shadow) price of cigarette *p* may include social image costs (e.g., of smoking being seen as ‘uncool’), and people may use this to correct their future behavior. As another example, Babcock and Hartman (2011) conduct a field experiment to examine the impact of financial incentives on attending sports gym. They find that participants whose peers are also treated are more likely to attend the gym. The potential problem here is that, unless we add some psychological or BE story of why these should matter in the given setting,^3^ this prediction does not in itself follow from the self-control models, therefore failing Test 2.

### Test 3: Empirical evidence

Table A2 in the Online Appendix summarizes a significant body of research on the potential usefulness of self-control devices in health settings.

*Voluntary use of self-control devices*. People may deliberately seek contracts that could be construed as implying a desire to constrain their choice set as predicted by self-control models (Halpern et al. 2012). For example, in a field experiment, Gine et al. (2010) investigated the effect of a voluntary commitment device on smoking cessation, i.e., Committed Action to Reduce and End Smoking (CARES). Smokers were offered a saving account in which after six months they are refunded subject to passing a nicotine test. Some smokers took up the scheme, with social pressure possibly having played a role. The smoking cessation rate was higher for the participants than for the control group, and the effects persisted in surprise tests one year later. However, given the well-documented frequent failure of consumers’ best intentions in health (e.g., London 2013), there is again a question of whether something else, such as social pressure, may have also been at work. Using US sports gyms data, DellaVigna and Malmendier (2006) find that consumers tend to enter fixed-term contracts and end up paying more per visit than they would have paid in fees for single visits. They interpret this as a form of overconfidence about either future self-control or future efficiency of gym visits.

It is not clear from existing evidence how much commitment one should make and it is also not clear how predictions from the self-control models would be verified, either in terms of learning (Ali 2011) or in terms of trade-off between flexibility and commitment (Amador et al. 2006). Moreover, if long run tastes change (Loewenstein et al. 2003), predicting the optimal commitment from the self-control model may not be appropriate.

*Policy interventions*. Non-voluntary commitment devices include governmental interventions, and there is a long strand of related literature (see Table A2 in the Online Appendix for a summary). For example, Charness and Gneezy (2009) conducted a field experiment with university students to evaluate the impact of financial incentives on attendance to a sports gym. Participants received money if they attended the gym as they were assigned. Charness and Gneezy found that this incentive scheme increased gym attendance even after the experimental intervention period, at least in the short term.

A problem in interpreting findings on the effect of price changes (or equivalent) on healthy behavior is that a more straightforward interpretation would be in terms of law of demand from basic microeconomics: as the price goes up, demand goes down. This would not explain an effect beyond the intervention period, and other theories would be needed for that, such as reinforcement theories from traditional behavioral psychology (e.g., Fantino and Logan 1979; Rachlin 1989), modern psychological habit system theories (e.g., Daw et al. 2011) or economic theories of rational addiction or habit formation (Becker and Murphy 1988; Rabin 2011). Self-control models however *also *do not explain effects beyond the intervention period, unless they are combined with other theories.

This brings us to the more general problem: these studies are all generally about the link between policy recommendations and empirical evidence (the link 6 of Fig. 1), but the empirical evidence is largely consistent with self-control models *as well as *a number of other models, and therefore is not the specific support of self-control models that we would like in terms of Test 3.^4^

### Summary

BE models of self-control *have *helped to bring self-control devices back in the policy debate, and, in this sense at least, they have proved practically important and useful. Self-control models have potential to pass Tests 1 and 2, but again there is an identification gap between models and evidence. There is also, in practice, often a need to combine self-control models with other kinds of behavioral economic models, such as ones on social interactions.

## Applying prospect theory to message framing

### The standard treatment of prospect theory and test 1

Prospect theory (Tversky and Kahneman 1981, 1992) innovatively combines dependence on a reference point, relative to which gains and losses can be identified; a value function implying that subjects are loss averse, i.e., they dislike losses more than they like gains; risk aversion in the domain of gains and risk lovingness in the domain of losses, as identified again in the value function; and probability weighting.^5^ Because of the modelling framework and of the parsimonious way it combines and draws implications from these features, it is plausible to assume that prospect theory passes Test 1. Indeed, it is referred to in the psychological research on health message framing starting from Rothman and Salovey (1997).

In relation to Test 2, health psychologists have drawn implications from prospect theory for policy makers. They have done so with a focus on the framing of the decision problem in terms of gains or losses and on the risk attitude differential features of the value function. A classic example was shown in experimental work by Tversky and Kahneman (1981): when people choose between two treatment programs framed in terms of the number of lives that will be lost, they risk the possibility of greater losses to avoid a certain loss; when the same programs are described in terms of the number of lives that will be saved, people become more conservative in their preferences. Hence they forego the opportunity for greater gains, in exchange for an alternative that provides a certain gain. Although the frame shifts in the two scenarios from lives lost to lives saved, the objective features of the proposed interventions remain constant (Tversky and Kahneman 1981).

In the health psychology research, the expectation has been that gain- and loss-framed appeals will be differentially persuasive for disease detection behaviors (such as mammography, HIV testing, or cholesterol screening) and disease prevention behaviors (such as healthy dieting, exercising, or dental hygiene), by virtue of the differences in the risk associated with those behaviors (Salovey and Wegener 2003; Schneider et al. 2001).^6^ The underlying idea is that prevention behavior tends to be perceived as entailing a reduction in the risk of the bad health outcome relative to the present health status, and so a gain frame encouraging risk aversion should lead to greater engagement in prevention behavior. Conversely, detection behaviors are typically perceived as involving a risk that illness may be discovered. A more risk loving attitude, as induced by a loss frame, would then be preferable.

### Test 2: Identifying predictions from prospect theory

This sub-section considers whether the typical prediction from prospect theory in a health context of prevention and detection activity passes Test 2: that is, is it generally the case that prospect theory implies that a loss frame is better than a gain frame for encouraging detection activity, and vice versa for prevention activity? As it turns out, the picture is more nuanced, and this can be shown even in a very simple application of prospect theory to our setting.

Assume for simplicity that there are two time periods, 1 and 2. There are two possible health outcomes, negative and positive. A negative health outcome occurs in period 2 with probability *p* in a state of the world in which prevention or detection activity has not taken place; if it has taken place, and purely to simplify the presentation below, we assume the negative health outcome never occurs. Prevention and detection behaviors take place in period 1. Prevention and detection activities have a financial and/or psychological cost of $z>0$. If detection activity takes place in period 1, in a gain frame there is a ‘pleasure’ $d>0$ of learning one is healthy, while in a loss frame there is a pain—*d* of learning that one is sick. Define *w*() as a weighted probability. We assume that the likelihood of the bad health outcome is not large and it is perceived and weighted by the person as not large relative to how the likelihood of a good health outcome is perceived:

#### Assumption 1

$$w(p)<<0.5<<w(1-p)$$.

Note that Assumption 1 permits the difference *w*(*p*)–*w*($$1-p)$$ to be smaller than that between *p*and ($$1-p)$$, i.e., it allows for overweighting of small probabilities; it plausibly assumes, however, that people will typically still be able to clearly identify what is more likely and what is not.

In a gain frame (+), the utilities of a positive and a negative health outcome in period 2 are perceived as *x* and 0, respectively, with $x>0$. In a loss frame (−), the utilities of a positive and a negative health outcome in period 2 are perceived as 0 and $-x$, respectively. We further assume that agents discount period 2 according to a discount factor $\delta \ge 0$, and that utility is additively separable between periods 1 and 2. Agents follow prospect theory and, in the absence of detection and prevention activity, we can write their utility function as:

- $U_+ =\frac{w(1-p)v(x)}{1+\delta }$ in a gain frame
- $U_- =\frac{w(p)v(-x)}{1+\delta }$ in a loss frame,

where $$U_+ $$and $$U_- $$are an increasing function of *w*() and *v*(); *v*()is the monotonically increasing prospect theory value function, and, for simplicity and without loss of generality, $$v(0)=0,v(x)>0,v(d)>0.$$ Our second assumption reflects the loss aversion and differential risk attitude in the domain of losses that is highlighted in the usual applications of prospect theory, with $$k_x>$$ 0 and $$k_d>$$ 0 being coefficients embodying the difference in valuation if *x*is perceived in the domain of losses rather than in that of gains:

#### Assumption 2

$$\left| {v(-x)} \right| =v(x)+k_x $$ and $$\left| {v(-d)} \right| =v(d)+k_d $$.

**Fig. 2:**
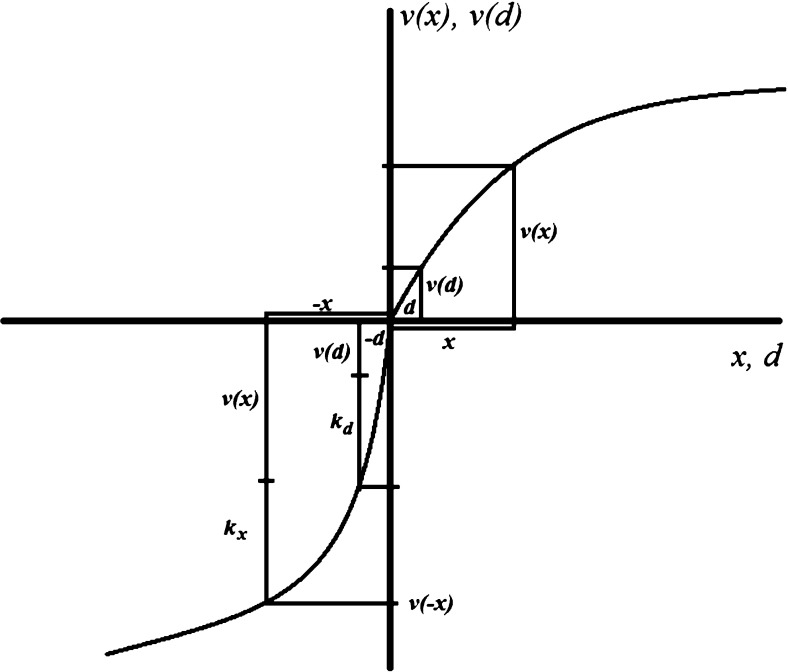
A prospect theory value function

Figure 2 shows a value function as an example.

*Prevention behavior*. In a gain frame, the agent will engage in prevention behavior in period 1 if the expected discounted gains from prevention are higher than the prevention cost:

3 $$\begin{aligned} \frac{w(1-p)v(x)}{1+\delta }-z>0. \end{aligned}$$

In a loss frame, the agent will engage in prevention behavior if the expected discounted avoided loss from prevention is higher than the prevention cost:

4 $$\begin{aligned} -\frac{w(p)v(-x)}{1+\delta }-z=\frac{w(p)[v(x)+k_x ]}{1+\delta }-z>0. \end{aligned}$$

If we restrict ourselves to Assumption 2 alone, $k_x$ implies that Eq. (4) is more likely to be satisfied than Eq. (3): intuitively, in a loss frame, the risk of a loss should naturally lead the loss averse person to engage in more prevention behavior to avoid the loss than if there is the possibility of a gain in a gain frame. Note however that, as long as the probability of the negative health outcome is small and remains perceived reasonably as such (Assumption 1), it is reasonable to hypothesize that $w(1-p)v(x)>w(p)v(x)+k_x$. If Assumption 1 does not hold, a negative frame may be roughly as good or even better than a gain frame. However, if Assumption 1 holds, a gain frame will generally be better for encouraging prevention, if not for the reason normally used.

*Detection behavior*. In comparing detection behavior with prevention behavior, there is an additional term to be considered, namely the psychological value *v*(*d*)in period 1 from knowing about a negative health outcome in period 2. In a gain frame, Eq. (3) now becomes:

5 $$\begin{aligned} \frac{w(1-p)v(x)}{1+\delta }-z-w(1-p)v(d)=w(1-p)\left[ {\frac{v(x)}{1+\delta }-v(d)} \right] -z>0 \end{aligned}$$

while Eq. (4) for a loss frame becomes:

6 $$\begin{aligned} -\frac{w(p)v(-x)}{1+\delta }-z+w(p)v(-d)=w(p)\left[ {\frac{v(x)+k_x }{1+\delta }-v(d)-k_d } \right] -z>0. \end{aligned}$$

Predictions here are generally ambiguous: while (6) could hold while (5) does not, implying that a loss frame is better in encouraging detection behavior, it is also entirely possible that (5) holds while (6) does not. Assume that (5) holds; Eq. (6) then may not hold if $k_x /(1+\delta )-k_d <0,$ i.e., depending on the degree of intertemporal discounting and on the precise shape of the value function in the loss domain relative to that in the gain domain.

### Test 3: considering empirical evidence on health message framing

We now move on to Test 3: how specific predictions of prospect theory fit with evidence on health message framing. Virtually all of this work has been carried out in a randomized experimental study design, as opposed to an observational one. A major difference among existing studies is the outcome variables, which comprise attitudes, intentions^7^ or actual behavior. We focus on behavior, as this is what prospect theory predictions are about. Table A3a in the Online Appendix summarizes a non-exhaustive list of primary studies on prevention behavior, most of which focus on oral health and physical activity promotion; and Table A3b in the Online Appendix assembles studies on detection behavior, of which a majority is on breast cancer screening. All of the studies in these tables use behavioral measures as their relevant outcomes.

*Prevention behaviors*. The studies covering prevention behaviors include a majority of studies on prevention of skin cancer, oral health problems and on the promotion of physical activity, while most of the detection behavior studies relate to breast cancer screening, followed by skin cancer screening. We list a few studies where *p*and as a result *v*(*p*) may be perceived as high: Knapp (1991) and Mann et al. (2004) are about oral health, and cavities may be seen as likely if oral health measures are not undertaken; in Richardson et al. (2004), the probability of HIV contagion may be perceived as high by the HIV-positive subject sample that was used; and in Trupp et al. (2011), again with a sample of patients. Other than Mann et al. (2004), where there is no aggregate effect of framing, in the other three cases a loss frame was superior in inducing prevention behavior (Knapp 1991; Trupp et al. 2011); these results are consistent with the prospect theory once Assumption 1 is relaxed (see Sect. 5.2). Gallagher and Updegraff (2011) explicitly look at the perceived probability *v*(*p*) and find the superiority of a loss frame when the perceived probability of breast cancer is average or high.

Where Assumption 1 is more likely to be satisfied, the picture is different. Detweiler et al. (1999) is an influential early study exploring the impact of message framing on skin cancer prevention was. They recruited a demographically and economically fairly diverse sample of 217 adult beach-goers in southern New England. Participants were given a brochure containing the framing manipulation as well as general information about skin cancer. A strong gain-framed advantage was found for the behavioral measure employed (requests for free sunscreen with protection factor 15), though the lack of follow up allowed no assessment of any sustained behavior change effect. Rothman et al. (1993) also found the superiority of a gain frame with a behavioral measure in relation to skin cancer prevention. In three out of six studies on physical activity or healthy diet reported in Table A3a in the Online Appendix, a gain frame was superior at least to some degree in three studies, with no significant effect of the frame in the other three (more detail can be found in the table).

*Detection behaviors*. Breast cancer screening has thus far been the most frequently examined detection behavior in this field. While some studies supported the original Rothman and Salovey (1997) prediction of loss-framed messages outperforming gain-framed ones when it comes to detection behaviors, other studies did not find significant difference; while one study in the context of smoking prevention found gain-framed messages outperforming loss-framed ones (see Table A3b in the Online Appendix for more detail). As an example, Finney and Iannotti (2002) explored an intervention aimed at increasing women’s adherence to recommendations for annual mammography screening. The intervention involved sending out one of three reminder letters (positive frame, negative frame, or standard hospital prompt) to 929 randomly selected women who were due for mammography screening and had been identified as having either a positive or negative family history of breast cancer. There was no significant effect of a gain vs. loss frame.

*Meta-analyses*. The discussion above has been selective, but, fortunately, a number of recent meta-analyses have been undertaken to examine the considerable empirical evidence base more systematically (for example, O’Keefe and Wu 2012; Gallagher and Updegraff 2012). No relevant meta-analysis that we are aware of supports the Rothman and Salovey (1997) interpretations of prospect theory as such. The meta-analysis of interest is Gallagher and Updegraff (2012), as it is the only one that focuses exclusively on studies that measured behavioral outcomes rather than attitudes or intentions. It is consistent with what we predicted in Sect. 5.2 based on a proper application of prospect theory: overall, gain-framed messages were more effective for prevention behaviors than loss-framed messages, while there was no clear superiority of either framing approach in the case of detection behaviors. Therefore, a properly applied prospect theory broadly passes Test 3.

### Summary

Based on Test 1, prospect theory is a good candidate as a BE model that may matter for health outcomes. This section considered health message framing as an area of policy relevance where prospect theory can be applied. The traditional interpretation of prospect theory does not follow from a basic model applying prospect theory, and so such a traditional interpretation does not pass Test 2. It also does not pass Test 3, given the empirical evidence.

Once prospect theory is properly applied, there are no clear predictions for detection activities, nor should they be expected. The good news is that a gain frame encourages prevention activity, though this does not apply if the perceived probability of the bad health outcome is large enough.

## Conclusions

We have proposed a triple test to evaluate the usefulness of BE models for public health policy. Test 1 is about yielding specific predictions relative to standard economic models or psychological theories in terms of individual behaviors or reaction to incentives. Test 2 is about linking the specific predictions to specific public health settings. Test 3 is about validating the model with evidence. Where a test is not passed, in particular Tests 2 or 3, this may point to directions for needed further research.

We have illustrated our analysis by considering, in different ways, three cases where a plausible claim can be made that Test 1 is passed. We recognize the differences across the cases: for example, prospect theory is a single model (if in different versions), whereas the other two cases are more akin to families of models building on related intuitions. Social interaction models need to be decomposed in different motivations for social interactions to have specific predictions for health policy settings and pass Test 2. Social learning, social comparisons and self-esteem motivations and associated models make different predictions for health policy settings, and more work is needed, in relation particularly to Test 3, as the experimental evidence is limited and as based on the current non-experimental evidence it is not possible to differentiate among different motivations. The evidence in relation to self-control models is largely consistent with self-control models *as well as *a number of other models, and so more work is needed to pass Test 3.

We have considered prospect theory in the context of health message framing as our third application. The claimed policy messages from the theory do not seem to match the evidence. By a simple example model, we have shown how the reason is that the theory has been misapplied. Once this problem with Test 2 is fixed, and the correct predictions are drawn from the model, we find that, in broad agreement with the evidence, a gain frame has positive implications for welfare as it does encourage disease prevention activity, though this does not apply if the perceived probability of the bad health outcome is large enough.

We see our tests as being useful to identify how much health policy weight to assign to specific behavioral economic models; and, constructively, to verify what next steps would be most useful in further research.

## Electronic supplementary material

Below is the link to the electronic supplementary material.

Supplementary material 1 (docx 35 KB)

## Footnotes

1 For a methodological criticism of libertarian paternalism as implied by the behavioral ‘nudge’ agenda, see Sugden (2004) and for a recent debate on this criticism, see Schubert (2015) and Sugden (2015).

2 A reader noted that these can be more general tests for evaluating the policy usefulness of models. While this may or may not be the case, this paper follows a more focused and practical approach.

3 Babcock and Hartman provide several alternative explanations of their findings: (i) complementarities in the utility; (ii) imitation; and (iii) information exchange among peers.

4 Burger and Lynham’s (2009) evidence on 70% of bettors explicitly referring to the need of a self-commitment device is obviously potentially more directly relevant evidence, though, as noted earlier, the same bettors ended up being money pumps for betting companies to take advantage ofbut the self-commitment device rarely worked.

5 We follow Barberis (2013) in this list and the cumulative prospect theory of Tversky and Kahneman (1992) in the treatment below. For broader developments in the study of decision making under risk in behavioral economics, see Starmer (2000).

6
Salovey and Wegener (2003), for instance, note that from “a prospect theory point of view, the perceived risk (of finding an abnormality) could make loss-framed messages more persuasive in promoting the detection behaviors. However [...] gain-framed messages might be more likely to facilitate performing prevention behaviors” (pp. 57–58).

7 See, e.g., Rothman et al. (1993) as a widely cited early study testing the effect of gain vs. loss framing on the *intention* to seek skin cancer screening.
