# Supplementary material for: A triple test for behavioral economics models and public health policy
Source: Theory Decis. 2017 Jul 18;83(4):513–33. doi: 10.1007/s11238-017-9625-9 (PMC6959397; doi:10.1007/s11238-017-9625-9)
Supplement: Supplementary file 1 — Supplementary material 1 (docx 35 KB) [file 11238_2017_9625_MOESM1_ESM.docx]

**Online Appendix for “A Triple Test for Behavioral Economics Models and Public Health Policy”**

Ryota Nakamura, Marc Suhrcke, and Daniel Zizzo (corresponding author: Newcastle University; [daniel.zizzo@newcastle.ac.uk](mailto:daniel.zizzo@newcastle.ac.uk))

*Theory & Decision*

This Online Appendix provides the summary of empirical findings relating to Sections 3, 4, and 5 of the main paper.

**Table A1: Social interactions**

| **Paper** | **Study design** | **Outcome** | **Subjects** | **Reference group** | **Claimed background economic theory** | **Key results** |
| --- | --- | --- | --- | --- | --- | --- |
| **A: Food consumption and body weight** | | | | | | |
| Anderson, 2009 | Statistical analysis of survey data | Perceived weight; weight goal | US High school students | Other students | N/A | Students in a heavier group perceive themselves as thinner than those in a thinner group. |
| Auld, 2011 | Analysis of survey data | Body Mass Index | US adults | People in the same country/state | Social comparison; Self-esteem | Country and state average weight are not associated with own weight. No clear evidence of country/state level socioeconomic characteristics on own weight. |
| Blanchflower et al., 2009 | Analysis of survey data | Overweight perceptions and diet/ Life satisfaction | Europeans | People in the same country, gender, and age group | Social comparison | Relative position of own weight in the peer group influences overweight perception, dieting, and wellbeing. |
| Burke and Heiland, 2007 | Modelling and simulation | Body Mass Index | US female adults | American female adults | Social comparison; Endogenous aggregate behaviour of social group | (Prediction:) as price of foods declines, body weight increases; then the norm level of weight increases, which leads to further increase of weight. |
| Carrell et al., 2011 | Analysis of survey data | Physical fitness (measured by fitness score) | Students at US Air Force Academy | Squadrons | N/A | There is a positive association between peers' fitness score in high school and own current fitness score. |
| Christakis and Fowler, 2007 | Analysis of survey data | Likelihood of being obese | US adults | Friends, siblings, spouse | N/A | There is a positive association between peer's obesity status and the likelihood of being obese. |
| Cohen-Cole and Fletcher, 2008 | Analysis of survey data | Body Mass Index | US junior high and high school students | Students in the same school | N/A | Peers' body weight has no effect on own weight (no network effect). |
| Croker et al., 2009 | Analysis of survey data/ experiment | Intention to eat fruits and vegetables | UK adults | Other people in the same country | N/A | Although individuals are less aware of importance of social norm compared to cost and health, giving information about norm influences the intention to eat fruit and vegetables. |
| Etilé, 2007 | Analysis of survey data | Ideal body weight; Food attitudes | French adults | People of same sex, occupation, and age | Social comparison; Identity theory | Social norm is associated with one's ideal body weight only for females who want to reduce weight (and not for other groups). Social norm does not predict food attitudes. |
| Fowler and Christakis, 2008 | Analysis of survey data | Body Mass Index | US adults | Named friends | N/A | There is a correlation between friends' body mass and own body mass. |
| Halliday and Kwak, 2009 | Analysis of survey data | Body Mass Index | US secondary school students | Ten nominated students | N/A | There is an association between peers' weight and own weight. The association is particularly strong among heavier students. |
| Pliner and Mann, 2004 | Laboratory experiment | Amount of food consumed/ food choice between palatable and unpalatable one | US female psychology students | Other participants | N/A | The amount of palatable food consumed is increased if participants are informed that other participants ate a lot of the same food. Food choice between palatable and unpalatable food is not affected by information of other participants' choice. |
| Rena et al., 2008 | Analysis of survey data | Body Mass Index | US secondary school students | Close friends | N/A | There is an association between peers' weight and own weight. Once the instrumental variable estimation approach is employed, the effect is significant only for female students. |
| Trogdon et al., 2008 | Analysis of survey data | Adolescent weight | US secondary school students | Students within the same grade in the same school | N/A | There is an association between peers' weight and own weight. The association is larger for female students. |
| Yakusheva et al., 2011 | Analysis of survey data | Weight gain after one year | US college students | Roommate | N/A | The amount of weight gained is lower if roommate's initial weight is lower. Female students adopt their roommates' weight reducing effort. |
| **B: Substance use** | | | | | | |
| Clark and Etile, 2006 | Analysis of survey data | Likelihood of smoking | British individuals | Partner | Social learning (also bargaining in marriage market) | There is a positive association between partner's smoking and own smoking. The association is due to assortative matching in marriage, rather than peer influence. |
| Clark and Loheac, 2007 | Analysis of survey data | Commission of risky behaviour (cigarette, alcohol, marijuana) | US junior high and high school students | Students in the same school | N/A | Peers' behaviour is correlated with own risky behaviour. |
| Duarte et al., 2013 | Analysis of survey data | Likelihood of smoking | Spanish adolescents | Students in the same class and school | N/A | The statistical significance of the peer effect is sensitive to the choice of estimator of standard error. |
| Fletcher, 2010 | Analysis of survey data | Likelihood of smoking | US secondary school students | Classmates | N/A | Larger proportion of classmates who smoke increases the likelihood of smoking. |
| Fletcher, 2012 | Analysis of survey data | Likelihood of alcohol drinking | US secondary school students | Classmates | N/A | Larger proportion of classmates who drink alcohol increases the likelihood of alcohol use. |
| Gaviria and Raphael, 2001 | Analysis of survey data | Likelihood of drug use, Alcohol, smoking, church attendance, high school drop-out | US students at tenth grade | Students in the same school | N/A | There is a correlation between peers' behaviour (drug use, alcohol, smoking, church attendance, and drop-out) and own behaviour. |
| Harris and López-Valcárcel, 2008 | Analysis of survey data | Likelihood of smoking | US young individuals | Siblings | Social learning | Presence of sibling who smokes in household increases the likelihood of smoking; presence of non-smoking sibling decreases the likelihood of smoking. |
| Jones, 1994 | Analysis of survey data | Smoking cessation; attempt to quit smoking | British adults | Other smokers within the household | N/A | Presence of other smokers in the same household is associated with lower probability of successful smoking cessation or probability of attempting to quit smoking. |
| Kawaguchi, 2004 | Analysis of survey data | Substance use (drug, cigarette, alcohol) | US teenagers | Students in the same grade | Social comparison | There is an association between peers' behaviour (drug use, alcohol, smoking) and own behaviour. |
| Krauth, 2005 | Analysis of survey data | Likelihood of smoking | Canadian youth | Close friends | N/A | Presence of peers who smoke is associated with the likelihood of smoking. |
| Krauth, 2006 | Analysis of survey data | Likelihood of smoking | US teenagers | Four same-sex friends | N/A | The association between presence of close friends who smoke and the likelihood of smoking is positive but nearly zero. |
| Krauth, 2007 | Analysis of survey data | Likelihood of smoking | US teenagers | Close friends | N/A | The association between presence of close friends who smoke and the likelihood of smoking is positive but nearly zero. |
| Lundborg, 2006 | Analysis of survey data | Teenage binge drinking, smoking, drug use | Swedish youth | Classmates | N/A | There is an association between peers' behaviour (drug use, alcohol, smoking) and own behaviour. |
| Nakajima, 2007 | Analysis of survey data | Likelihood of smoking | US students (6-12 grades) | Same school cohort (middle and high school) | Self-image | There is an association between prevalence peers who smoke and the likelihood of smoking. The effect is stronger in the same gender and race. |
| Norton et al., 1998 | Analysis of survey data | Use of alcohol and smoking | US upper elementary student | Students from same elementary school | N/A | There is an association between peers' behaviour (alcohol use and smoking) and own behaviour. Peer influence is more important than peer selection effect. |
| Powell et al., 2005 | Analysis of survey data | Likelihood of smoking | US high school students | Other students in the same high school | N/A | There is an association between prevalence peers who smoke and the likelihood of smoking. |
| Svensson, 2010 | Analysis of survey data | Alcohol use and binge drinking | Swedish youth | Students in the same school | Social comparison; social learning; pay-off interaction | Prevalence of peers who frequently drinks alcohol and commit binge drinking is associated with one’s own alcohol and binge drinking. |
| **C: Other outcomes** | | | | | | |
| Bramoullé et al., 2009 | Analysis of survey data | Consumption of recreational services | US secondary school students | Best friends | N/A | Recreational activities by friends increase one's own use of recreational services. |
| Costa-Font and Jofre-Bonet, 2013 | Analysis of survey data | Suffering from anorexia | European young females | Self-image on own weight; people with same education, age, rural/urban, and region | Social comparison; Identity theory | Peer's body mass is negatively associated with likelihood of being anorexia. |
| Sorensen, 2006 | Analysis of survey data | Health care plan choice | University of California staff | Colleagues in the same department | Social learning | Information about peers' choice influences one's own choice of health care plan. |
| Lindbeck et al. 2016 | Analysis of survey data | Sick leave | Members of the Swedish sick-pay insurance system | Neighbourhood | Social scrutiny, Stigma, Social learning | Greater average days of sick leave in the neighbourhood increases the days of own sick leave. |

**Table A2: Self control devices**

| **Paper** | **Study design** | **Outcome** | **Subjects** | **Self-control device** | **Intervention period** | **Key results** |
| --- | --- | --- | --- | --- | --- | --- |
| **A: Food consumption, physical activity, and body weight** | | | | | | |
| Augurzky et al. 2012 | Field experiment | Weight reduction | Obese patient in Germany | Conditional cash transfer | 4 months | Financial incentive was effective to achieve weight loss target |
| Babcock and Hartman, 2011 | Field experiment | Gym attendance | US university students | Conditional cash transfer and peer effects | 4 weeks | Gym attendance for the incentivized group increased when their peers were also incentivized |
| Burger and Lynham, 2009 | Analysis of survey data | Weight reduction | UK adults | Self-commitment betting for successful weight loss with a bookmaker | N/A (Observational study; Betting period vary across bettors) | Weight loss was rarely successful |
| Cawley and Price, 2013 | Analysis of non-random trial data | Weight reduction | Company employees | Fixed payment and money deposit contract | 4 quarters | Financial incentive was associated with only modest weight reduction |
| Charness and Gneezy, 2009 | Field experiment | Gym attendance | US university students | Conditional cash transfer | 4 Weeks; and 7 weeks follow-up for Study 1 and 13 weeks follow-up for Study 2 | Financial incentive was effective and also the effect was sustainable |
| Della-Vigna and Malmendier, 2006 | Analysis of survey data | Gym attendance | US adults | Contracts with sports gyms for monthly fixed free | N/A | Those who made fixed term contract tended to overestimate their will power |
| Goldhaber-Fiebert et al., 2011 | Field experiment | Length of contract with sports gym | US adults | Longer default contract periods | N/A | Setting a longer contract length as a default option is associated with longer actual contract |
| Royer et al., 2015 | Field experiment | Gym attendance | Employees of a Fortune 500 company (US) | Conditional cash transfer and self-commitment deposit (refunded subject to gym attendance) | 4 weeks for financial incentive treatment; 8 weeks for commitment deposit, with 12 months follow-up | Gym attendance was increased, and the effect was maintained but diminished in the post-treatment periods |
| Volpp et al., 2008 | Field experiment | Weight reduction | US adults | Lottery incentive; or deposit contract | 16 weeks; and additional 6 months for selected participants only. Follow-up tests were conducted after 7-8 months of the intervention | The intervention increased successful weight reduction in the treatment period, but the effect diminished in the follow up test |
| Wisdom et al., 2010 | Field experiment | Food choice at a cafeteria | Customers of a US fast-food restaurant | Providing calorie information; making healthy option convenient | N/A | The interventions were largely effective |
| **B: Other outcomes** | | | | | | |
| Gine et al., 2010 | Field experiment | Smoking cessation | Clients of a bank in Philippines | Self-commitment saving account (refunded subject to successful smoking cessation) | 6 weeks, and a follow-up test after 12 months of the treatment | The use of the commitment saving account was associated with successful smoking cessation; the effect was sustainable |
| Kohler and Thornton, 2012 | Field experiment | HIV prevention | Adults in rural Malawi | Conditional cash transfer for maintaining HIV status | Around one year (two rounds of HIV tests were conducted) | Conditional cash transfer had no effect on the HIV status |

**Table A3a: Selected experimental studies testing message framing in prevention behavior**

| **Paper** | **Outcome /behaviour** | **Subjects** | **Intervention content** | **Intervention delivery** | **Key results** |
| --- | --- | --- | --- | --- | --- |
| **A: Skin Cancer Prevention** | | | | | |
| Detweiler et al., 1999 | Skin cancer prevention | Beach visitors (US) | Gain framed messages (including value of skin protection) vs. loss framed messages | Brochure | 18% increase in collection of sunscreens |
| Rothman et al., 1993 | Skin cancer prevention | Undergraduate students (US) | Gain and loss framed messages | Brochure | Gain framed messages more effective in making women buy sun protection crème |
| **B: Physical activity and healthy diet** | | | | | |
| Bannon and Schwartz, 2006 | Healthy diet promotion | Elementary school children, mean age 5 (3 classrooms) (US) | (a) A gain-framed nutrition message (i.e. the positive benefits of eating apples) ; (b) a loss-framed message (i.e. the negative consequences of not eating apples); (c) control scene (children playing a game) | Video (commercial) | Among the children who saw one of the nutrition message videos, 56% chose apples rather than animal crackers; in the control condition only 33% chose apples. (no significant difference between gain and loss frame) |
| Gallagher and Updegraff, 2011 | Physical activity | "Mostly sedentary" undergraduate students (US) | Gain and loss framed messages (incl value of more PA) with intrinsic and extrinsic exercise outcomes | Article on exercise | Loss framed messages not significantly more effective in promoting PA |
| Jones et al., 2003 | Physical activity | Introductory psychology students (US) | Gain and loss framed message (+ background reading from credible / less credible source) | Read a message (after background material from credible/less credible source) | Gain framed messages from a credible source more effective in promoting exercise than other interventions |
| Jones et al., 2004 | Physical activity | Introductory psychology students (US) | Gain framed messages attributed to credible / non-credible source | Read messages | No significant effects |
| Latimer et al., 2008 | Physical activity | Sedentary, healthy callers to the US National Cancer Institute’s Cancer Information Service | Gain-, loss-, or mixed-framed messages | Reading material in print | Gain frame messages generally significantly more effective than comparators |
| Lawatsch, 1990 | Healthy diet promotion | Pre-school children (US) | Gain-framed, loss-framed, control | Reading of adjusted fairy tales | Modest significant advantage of gain frame message |
| **C: Oral health** | | | | | |
| Knapp, 1991 | Oral health | Elementary school children age 10-12 (US) | Gain or loss framed messages; control group (basic information, no mention of consequences) | Audio taped slide show | Loss framed messages more effective than gain framed and standard message |
| Mann et al., 2004 | Oral health (flossing) | Undergraduate students (US) | Gain or loss framed messages | Articles to read | No significant effect of framing; but when given a loss-framed message, avoidance-oriented people reported flossing more than approach-oriented people, and when given a gain-framed message, approach oriented people reported flossing more than avoidance-oriented people |
| **D: Other outcomes** | | | | | |
| Gerend and Cullen, 2008 | Aalcohol | College students (US) | Gain framed message | Read a message | Gain-framed message significantly effective in reducing (self-reported alcohol use) compared to loss framed message (but only for short term consequences) |
| Richardson et al., 2004 | Safe sex prevention | HIV positve, sexually active prior to enrollment (US) | Gain-framed, loss-framed, control | Prevention counselling from medical providers supplemented with written information | Significant effects in the loss-framed education only |
| Schneider et al., 2001 | Smoking prevention | Undergraduate students (US) | Gain, loss framed | Video | Gain framed messages more effective than loss framed |
| Trupp et al., 2011 | Adherence to continuous positive airway pressure (CPAP therapy) to prevent obstructive sleep apnea [OSA] | Adults with a history of CVD who were newly diagnosed with OSA (US) | Loss and gain framed messages about CPAP | Video | CPAP use was greater in the group receiving negative message framing |

**Table A3b: Selected experimental studies testing message framing in detection behavior**

| **Paper** | **Outcome/behaviour** | **Subjects** | **Intervention content** | **Intervention delivery** | **Key results** |
| --- | --- | --- | --- | --- | --- |
| **A: Breast cancer screening** | | | | | |
| Banks et al., 1995 | Breast cancer screening | Woman workers aged > 40 with history of poor utilization of screening (US) | Loss framed messages (emphasizing risk of not being screened) vs. gain framed messages | Video | 14.7% increase in uptake of mammography |
| Consedine et al., 2007 | Breast cancer screening | Low-income, low-screening women (US) | Loss, gain, or empowerment frame telephone intervention and re-contacted at 6 and 12 months. | Phone | No main effect for framing condition, |
| Finney and Iannotti, 2002 | Breast cancer screening | Women due for screening and either positive or negative family history of breast cancer; in rural area not for profit hospital (US) | Loss framed messages (emphasizing risk of not being screened) vs. gain framed messages | Reminder letter | No significant differences |
| Gallagher et al., 2011 | Breast cancer screening | Women recruited from an inner city hospital, non-adherent to guidelines for receiving annual screening mammograms (US) | Gain- or loss-framed message about the importance of mammography | Video | Women with average and higher levels of perceived susceptibility for breast cancer were significantly more likely to report screening after viewing a loss-framed message compared to a gain-framed message. No such framing effects for women with lower levels of perceived susceptibility. |
| Lalor and Hailey, 1990 | Breast cancer screening | Undergraduate women, self-reporting to do breast self-examinations (US) | Loss vs. gain framed messages | Written (pamphlets) | No significant differences |
| Lerman et al., 1992 | Breast cancer screening | Female HMO members aged 50-74 with abnormal mammogram (US) | Loss framed messages (emphasizing risk of not being screened) vs. gain framed messages | Written | No significant differences |
| Williams et al., 2001 | Breast cancer screening | Women randomly selected from telephone directory (Australia) | Loss framed, gain framed and neutral messages in brochure | Telephone contact + brochure | Loss-framed brochures led to significantly greater change in a positive direction than did gain-framed brochures, which were more effective than were neutral (no frame) brochures |
| **B: Other outcomes** |  |  |  |  |  |
| Apanovitch et al., 2003 | HIV testing | Low income ethnic minority women (US) | Two gain framed and two loss framed videos to motivate HIV testing | Video | Loss-framed messages only more effective than gain-framed messages for people who were uncertain about what the outcome of the test. Those certain that the test would not find the presence of HIV, gain-framed messages were more effective in promoting testing than loss-framed messages. |
| Gintner et al., 1987 | Blood pressure screening | Undergraduates with and without a hypertensive parent (US) | Loss or gain framed messages about hypertension and the importance of early detection | Printed material handed out | Gain frame found more than twice effective with subjects with history of parental hypertension |
| Lauver and Rubin, 1990 | Cervical smears testing | Women with abnormal smears and no previous colposcopy (US) | Loss framed messages (emphasizing risk of not being screened) vs. gain framed messages | Telephone contact + written | 5.2% increase in uptake of colposcopy |
| Myers et al., 1991 | Colorectal screening | Men aged 50-74, members of HMO (US) | Loss framed messages (emphasizing risk of not being screened) vs. gain framed messages | Telephone contact + written | 3.4% increase in adherence t screening |
| Park et al., 2010 | Type 2 diabetes screening | High risk individuals aged 40-69 years in two general practices (UK) | Loss and gain framed messages in an invitation to screening for type 2 diabetes | Written invitation | No significant differences in attendance to the screening between the loss and gain frame arms |

**References for Table A1, A2, A3a, and A3b**

Anderson, L., 2009. The trend in obesity: The effect of social norms on perceived weight and weight goal. Binghamton University

Apanovitch, A.M., McCarthy, D., Salovey, P., 2003. Using message framing to motivate HIV testing among low-income, ethnic minority women. Health Psychology 22, 60-67

Augurzky, B., Bauer, T.K., Reichert, A.R., Schmidt, C.M., Tauchmann, H., 2012. Does money burn fat? Evidence from a randomized experiment. IZA Discussion Paper No. 6888

Auld, M.C., 2011. Effect of large-scale social interactions on body weight. Journal of Health Economics 30, 303-316

Babcock, P., Hartman, J., 2011. Networks and workouts: treatment status specific peer effects in a randomized field experiment. University of California, Santa Barbara

Banks, S.M., Salovey, P., Greener, S., Rothman, A.J., Moyer, A., Beauvais, J., Epel, E., 1995. The effects of message framing on mammography utilization. Health Psychology 14, 178-84

Bannon, K., Schwartz, M.B., 2006. Impact of nutrition messages on children's food choice: pilot study. Appetite 46, 124-9

Blanchflower, D.G., Van Landeghem, B., Oswald, A.J., 2009. Imitative obesity and relative utility. Journal of the European Economic Association 7, 528-538

Bramoullé, Y., Djebbari, H., Fortin, B., 2009. Identification of peer effects through social networks. Journal of Econometrics 150, 41-55

Burger, N., Lynham, J., 2009. Betting on weight loss … and losing: personal gambles as commitment mechanisms. Applied Economics Letters 17, 1161-1166

Burke, M.A., Heiland, F., 2007. Social dynamics of obesity. Economic Inquiry 45, 571-591

Camerer, C., Loewenstein, G., Rabin, M., 2004. Advances in behavioral economics. Princeton University Press, Princeton and Oxford

Carrell, S.E., Hoekstra, M., West, J.E., 2011. Is poor fitness contagious?: evidence from randomly assigned friends. Journal of Public Economics 95, 657-663

Cawley, J., Price, J.A., 2013. A case study of a workplace wellness program that offers financial incentives for weight loss. Journal of Health Economics 32, 794-803

Charness, G., Gneezy, U., 2009. Incentives to exercise. Econometrica 77, 909-931

Christakis, N.A., Fowler, J.H., 2007. The spread of obesity in a large social network over 32 years. New England Journal of Medicine 357, 370-379

Clark, A.E., Etilé, F., 2006. Don’t give up on me baby: spousal correlation in smoking behaviour. Journal of Health Economics 25, 958-978

Clark, A.E., Lohéac, Y., 2007. “It wasn’t me, it was them!” Social influence in risky behavior by adolescents. Journal of Health Economics 26, 763-784

Cohen-Cole, E., Fletcher, J.M., 2008. Is obesity contagious? Social networks vs. environmental factors in the obesity epidemic. Journal of Health Economics 27, 1382-1387

Consedine, N.S., Horton, D., Magai, C., Kukafka, R., 2007. Breast screening in response to gain, loss, and empowerment framed messages among diverse, low-income women. Journal of Health Care for the Poor and Underserved 18, 550-66

Costa-Font, J., Jofre-Bonet, M., 2013. Anorexia, body image and peer effects: evidence from a sample of european women. Economica 80, 44-64

Croker, H., Whitaker, K.L., Cooke, L., Wardle, J., 2009. Do social norms affect intended food choice? Preventive Medicine 49, 190-193

DellaVigna, S., Malmendier, U., 2006. Paying not to go to the gym. American Economic Review 96, 694-719

Detweiler, J.B., Bedell, B.T., Salovey, P., Pronin, E., Rothman, A.J., 1999. Message framing and sunscreen use: gain-framed messages motivate beach-goers. Health Psychology 18, 189-96

Duarte, R., Escario, J.J., Molina, J.A., 2013. Are estimated peer effects on smoking robust? evidence from adolescent students in Spain. Empirical Economics, 1-13

Etilé, F., 2007. Social norms, ideal body weight and food attitudes. Health Economics 16, 945-966

Finney, L.J., Iannotti, R.J., 2002. Message framing and mammography screening: a theory-driven intervention. Behavioral Medicine 28, 5-14

Fletcher, J., 2012. Peer influences on adolescent alcohol consumption: evidence using an instrumental variables/fixed effect approach. Journal of Population Economics 25, 1265-1286

Fletcher, J.M., 2010. Social interactions and smoking: evidence using multiple student cohorts, instrumental variables, and school fixed effects. Health Economics 19, 466-484

Fowler, J.H., Christakis, N.A., 2008. Estimating peer effects on health in social networks: a response to Cohen-Cole and Fletcher; and Trogdon, Nonnemaker, and Pais. Journal of Health Economics 27, 1400-1405

Gallagher, K.M., Updegraff, J.A., 2011. When 'fit' leads to fit, and when 'fit' leads to fat: how message framing and intrinsic vs. extrinsic exercise outcomes interact in promoting physical activity. Psychology & Health 26, 819-34

Gallagher, K.M., Updegraff, J.A., Rothman, A.J., Sims, L., 2011. Perceived susceptibility to breast cancer moderates the effect of gain- and loss-framed messages on use of screening mammography. Health Psychology 30, 145-52

Gaviria, A., Raphael, S., 2001. School-based peer effects and juvenile behavior. Review of Economics and Statistics 83, 257-268

Gine, X., Karlan, D., Zinman, J., 2010. Put your money where your butt is: a commitment contract for smoking cessation. American Economic Journal: Applied Economics 2, 213-235

Gintner, G.G., Rectanus, E.F., Achord, K., Parker, B., 1987. Parental history of hypertension and screening attendance: effects of wellness appeal versus threat appeal. Health Psychology 6, 431-44

Goldhaber-Fiebert, J.D., Blumenkranz, E., Garber, A.M., 2010. Commiting to exercise: contract design for virtuous habit formation. NBER Working Paper No. 16624

Grend, M.A., Cullen, M., 2008. Effects of message framing and temporal context on college student drinking behavior. Journal of Experimental Social Psychology 44, 1167-1173

Halliday, T.J., Kwak, S., 2009. Weight gain in adolescents and their peers. Economics & Human Biology 7, 181-190

Harris, J.E., González López-Valcárcel, B., 2008. Asymmetric peer effects in the analysis of cigarette smoking among young people in the United States, 1992–1999. Journal of Health Economics 27, 249-264

Jones, A.M., 1994. Health, addiction, social interaction and the decision to quit smoking. Journal of Health Economics 13, 93-110

Jones, L.W., Sinclair, R.C., Courneya, K.S., 2003. The effects of source credibility and message framing on exercise intentions, behaviors, and attitudes: An integration of the elaboration likelihood model and prospect theory. Journal of Applied Social Psychology 33, 179-196

Jones, L.W., Sinclair, R.C., Rhodes, R.E., Courneya, K.S., 2004. Promoting exercise behaviour: an integration of persuasion theories and the theory of planned behaviour. British Journal of Health Psychology 9, 505-21

Kawaguchi, D., 2004. Peer effects on substance use among American teenagers. Journal of Population Economics 17, 351-367

Knapp, L.G., 1991. Effects of type of value appealed to and valence of appeal on children's dental health behavior. Journal of Pediatric Psychology 16, 675-86

Kohler, H.-P., Thornton, R.L., 2012. Conditional cash transfers and HIV/AIDS prevention: Unconditionally promising? World Bank Economic Review 26, 165-190

Krauth, B.V., 2005. Peer effects and selection effects on smoking among Canadian youth. Canadian Journal of Economics 38, 735-757

Krauth, B.V., 2006. Simulation-based estimation of peer effects. Journal of Econometrics 133, 243-271

Krauth, B.V., 2007. Peer and selection effects on youth smoking in California. Journal of Business and Economic Statistics 25, 288-298

Lalor, K.M., Hailey, B.J., 1990. The effects of message framing and feelings of susceptibility to breast cancer on reported frequency of breast self-examination. International Quarterly of Community Health Education 10, 183-192

Latimer, A.E., Rench, T.A., Rivers, S.E., Katulak, N.A., Materese, S.A., Cadmus, L., Hicks, A., Keany Hodorowski, J., Salovey, P., 2008. Promoting participation in physical activity using framed messages: an application of prospect theory. British Journal of Health Psychology 13, 659-81

Lauver, D., Rubin, M., 1990. Message framing, dispositional optimism, and follow-up for abnormal Papanicolaou tests. Research in Nursing & Health 13, 199-207

Lawatsch, D.E., 1990. A comparison of two teaching strategies on nutrition knowledge, attitudes and food behavior of preschool children. Journal of Nutrition Education 22, 117-123

Lerman, C., Ross, E., Boyce, A., Gorchov, P.M., McLaughlin, R., Rimer, B., Engstrom, P., 1992. The impact of mailing psychoeducational materials to women with abnormal mammograms. American Journal of Public Health 82, 729-30

Lindbeck, A., Palme, M., Persson, M., 2016. Sickness absense and local benefit cultures. Scandinavian Journal of Economics 118 (1) 49-78

Lundborg, P., 2006. Having the wrong friends? Peer effects in adolescent substance use. Journal of Health Economics 25, 214-233

Mann, T., Sherman, D., Updegraff, J., 2004. Dispositional motivations and message framing: a test of the congruency hypothesis in college students. Health Psychology 23, 330-4

Myers, R.E., Ross, E.A., Wolf, T.A., Balshem, A., Jepson, C., Millner, L., 1991. Behavioral interventions to increase adherence in colorectal cancer screening. Medical Care 29, 1039-50

Nakajima, R., 2007. Measuring peer effects on youth smoking behaviour. Review of Economic Studies 74, 897-935

Norton, E.C., Lindrooth, R.C., Ennett, S.T., 1998. Controlling for the endogeneity of peer substance use on adolescent alcohol and tobacco use. Health Economics 7, 439-453

Park, P., Simmons, R.K., Prevost, A.T., Griffin, S.J., 2010. A randomized evaluation of loss and gain frames in an invitation to screening for type 2 diabetes: effects on attendance, anxiety and self-rated health. Journal of Health Psychology 15, 196-204

Pliner, P., Mann, N., 2004. Influence of social norms and palatability on amount consumed and food choice. Appetite 42, 227-37

Powell, L.M., Tauras, J.A., Ross, H., 2005. The importance of peer effects, cigarette prices and tobacco control policies for youth smoking behavior. Journal of Health Economics 24, 950-968

Renna, F., Grafova, I.B., Thakur, N., 2008. The effect of friends on adolescent body weight. Economics & Human Biology 6, 377-387

Richardson, J.L., Milam, J., McCutchan, A., Stoyanoff, S., Bolan, R., Weiss, J., Kemper, C., Larsen, R.A., Hollander, H., Weismuller, P., Chou, C.P., Marks, G., 2004. Effect of brief safer-sex counseling by medical providers to HIV-1 seropositive patients: a multi-clinic assessment. AIDS 18, 1179-86

Rothman, A.J., Salovey, P., Antone, C., Keough, K., Martin, C.D., 1993. The influence of message framing on intentions to perform health behaviors. Journal of Experimental Social Psychology 29, 408-433

Royer, H., Stehr, M., Sydnor, J., 2015. Incentives, Commitments, and Habit Formation in Exercise: Evidence from a Field Experiment with Workers at a Fortune-500 Company. American Economic Journal: Applied Economics 7, 51-84

Schneider, T.R., Salovey, P., Apanovitch, A.M., Pizarro, J., McCarthy, D., Zullo, J., Rothman, A.J., 2001. The effects of message framing and ethnic targeting on mammography use among low-income women. Health Psychology 20, 256

Sorensen, A.T., 2006. Social learning and health plan choice. RAND Journal of Economics 37, 929-945

Svensson, M., 2010. Alcohol use and social interactions among adolescents in Sweden: do peer effects exist within and/or between the majority population and immigrants? Social Science & Medicine 70, 1858-1864

Trogdon, J.G., Nonnemaker, J., Pais, J., 2008. Peer effects in adolescent overweight. Journal of Health Economics 27, 1388-1399

Trupp, R.J., Corwin, E.J., Ahijevych, K.L., Nygren, T., 2011. The impact of educational message framing on adherence to continuous positive airway pressure therapy. Behavioral Sleep Medicine 9, 38-52

Volpp, K., John, L., Troxel, A., Norton, L., Fassbender, J., Loewenstein, G., 2008. Financial incentive–based approaches for weight loss: a randomized trial. Journal of the American Medical Association 300, 2631-2637

Williams, T., Clarke, V., Borland, R., 2001. Effects of message framing on breast-cancer-related beliefs and behaviors: the role of mediating factors. Journal of Applied Social Psychology 31, 925-950

Wisdom, J., Downs, J.S., Loewenstein, G., 2010. Promoting healthy choices: Information versus convenience. American Economic Journal: Applied Economics 2, 164-178

Yakusheva, O., Kapinos, K., Weiss, M., 2011. Peer effects and the Freshman 15: Evidence from a natural experiment. Economics & Human Biology 9, 119-132
